# Editorial: Neurobiological basis of substance use disorders: new findings and perspectives

**DOI:** 10.3389/fnins.2025.1744297

**Published:** 2026-02-20

**Authors:** Cristiane Aparecida Favoretto, Cássio Morais Loss, Valentina Vozella, Fábio Cardoso Cruz

**Affiliations:** 1 Department of Psychiatry and Human Behavior, University of Mississippi Medical Center, Jackson, MS, United States; 2 Stiles-Nicholson Brain Institute, Charles E. Schmidt College of Medicine, Florida Atlantic University, Jupiter, FL, United States; 3 Department of Translational Medicine, The Scripps Research Institute, La Jolla, CA, United States; 4 Molecular and Behavioral Neuroscience Laboratory, Pharmacology Department, Universidade Federal de São Paulo, São Paulo, Brazil

**Keywords:** impulsivity, neural circuits, neural targets, risk factors, substance use disorders

Substance use disorders (SUDs) represent a major public health issue that affects millions of individuals worldwide. Multiple factors (*e.g*., genetic, psychosocial, and environmental) are involved in the development of SUDs, unraveling the complexity to find effective treatments for all individuals. While different neurotransmitter systems, neural circuits, and pathways have been investigated as potential targets for intervention, the neurobiological substrates of SUDs remain only partially understood. This Research Topic gathers studies that broaden our understanding of the neural mechanisms for the onset, development, and maintenance of SUDs, as well as risk factors, comorbidities, and modulators associated with these disorders. Below, we summarize the overall aims and findings of the original research and review articles that compose this Research Topic.

This Research Topic includes three reviews. The first, by Amancio-Belmont et al., discussed how cannabinoid type 1 receptors (CB1) seem to represent one of the mechanisms underlying SUDs, through their regulation of goal-directed behaviors, reward sensitivity, and inhibitory control. The expression pattern of CB1 appears to vary in an age-dependent manner in different brain regions (Amancio-Belmont et al., 2017; Romero-Torres et al., 2023), which may be related to changes in impulse control during specific ontogenetic periods, such as adolescence. Besides the most explored role of the prefrontal cortex in behavior regulation and executive control, this review explores studies reporting the involvement of CB1 signaling in modulating behavioral inhibition in other regions, including the hippocampus and cerebellum (*e.g*., Abela and Chudasama, 2014; Ortiz et al., 2015). Modulation of CB1 signaling through pharmacological CB1 antagonism/inverse agonism has been tested, but there is a challenge in avoiding undesired serious psychological adverse effects, such as depressive symptoms.

In the second review, Van Doorn et al. addressed the role of the neuropeptide pituitary adenylate cyclase-activating peptide (PACAP) on neuropsychiatric disorders and SUDs. The authors explored sex-dimorphic characteristics at the molecular, brain regional, and behavioral levels. Stress, post-traumatic stress disorder (PTSD), anxiety, depression, schizophrenia, as well as multiple contexts (*e.g*., nicotine, ethanol, and cocaine) and stages of addiction (intoxication, withdrawal, and preoccupation/anticipation) are among the subjects discussed. According to the authors, understanding the sex-specific differences in the PACAP system offers a foundation for future studies aimed at developing tailored interventions for addressing SUDs.

The third is a mini-review by Juliano et al., which explores the profound neurobiological relationship between stress exposure and SUDs development, highlighting how both conditions share disruptions in major brain circuits and exhibit features like neuroinflammation and oxidative stress. Early-life stress is identified as a critical risk factor that increases vulnerability to SUDs development and relapse by mediating long-term changes in the reward and stress pathways. Finally, the authors suggest that cellular stress and genetic factors are promising targets for therapeutic interventions.

The original research authored by Favoretto et al. also examined the effect of stress on ethanol intake and the underlying mechanisms. Specifically, authors investigated how predator odor stress interacts with chronic intermittent ethanol (CIE) vapor exposure to alter: CIE-induced escalation in ethanol intake, plasma corticosterone levels, and the expression or co-localization of CB1 receptors, D1-medium spiny neurons (D1-MSNs), and Fos neuronal activation markers in the nucleus accumbens (NAc) and dorsal striatum. They found that predator odor stress accelerated CIE-induced escalation of ethanol intake but CIE did not potentiate corticosterone response. The CIE and predator odor stress effects on neuronal circuitry were distinct: CIE broadly increased activation in the NAc across various neuronal subtypes (enhanced proportion of *Fos*+, dual *Cnr1/Fos*+ and *Drd1/Fos*+, and triple *Cnr1/Drd1/Fos*+ cells), whereas repeated stress reduced the percentage of *Fos*+ and triple-labeled *Cnr1/Drd1/Fos*+ cells in the NAc, but not dorsal striatum, potentially highlighting a unique role for this neuronal subpopulation in stress-related neural adaptations.

Also aiming to explore the neurobiological substrates of alcohol use disorders (AUD), Simões et al. tested the effects of 5-HT2A and 5-HT2C antagonists on the acquisition and expression of voluntary ethanol drinking. The authors used a two-bottle choice paradigm to determine the role of the serotonin 5-HT2A and 5-HT2C receptors on ethanol consumption and preference during an acquisition phase and during re-exposure to ethanol after abstinence periods. Two independent experiments using adult Swiss male mice as experimental subjects were performed, in which 5-HT2A and 5-HT2C antagonists (either alone or combined) were administered either before acquisition (Experiment 1) or during the abstinence period preceding re-exposure sessions (Experiment 2). The authors found that the 5-HT2A and 5-HT2C receptors differentially modulate the acquisition and expression of voluntary ethanol drinking in mice. Therefore, 5-HT2A and 5-HT2C receptor antagonists could represent promising pharmacological treatments for AUD; however, their efficacy and safety warrant further investigation.

Finally, transitioning from preclinical to a clinical perspective, the study by Lippard et al. utilized resting-state functional MRI to investigate the subjective and neural responses to placebo and ethanol administration in young adults diagnosed with bipolar disorder (BD) and healthy individuals. Individuals with BD reported greater subjective intoxication following both placebo and ethanol conditions, and exhibited increased connectivity between the NAc and the postcentral/supramarginal gyrus (within the sensorimotor network) during the placebo condition, compared to the pre-beverage baseline. Healthy subjects showed reduced connectivity between NAc and postcentral/supramarginal gyrus following placebo administration. Furthermore, higher anxiolytic effects under placebo conditions were correlated with augmented placebo-adjusted NAc connectivity (NAc-to-left postcentral/supramarginal gyrus), and ethanol use at the follow-up in individuals with BD, but not in the healthy individuals' group.

The collection of manuscripts in this Research Topic explores vulnerability factors and the intricate neurobiological mechanisms involved in SUDs. Overall, studies pointed to stress as an important risk factor for SUDs and the importance of considering individual differences, including sex and history of other disorders, for the development of tailored and improved treatment options for SUDs. In addition, the studies contained here point to the roles of specific neural targets, including the cannabinoid and serotonergic systems, as well as neural circuits/hubs such as the NAc, as possibly promising for future interventions.

Despite the convergences observed across these and other studies in the literature, the high heterogeneity of experimental protocols, species used, and specific characteristics of experimental subjects makes comparisons across studies in the SUDs field challenging. Moreover, limitations of studies frequently include underrepresentation of sex differences, translational gaps, reliance on cross-sectional imaging, and small sample sizes, and should be taken into consideration when making overgeneralizing statements about these complex, multifactorial disorders. Given that, researchers should consider synthesizing evidence using focused, low-bias approaches such as systematic reviews and meta-analyses, which allow assessment of effect sizes and the quality of the original studies.

The editors hope readers will find this Research Topic helpful in identifying gaps in the current understanding of these conditions and in gaining insights for future research that aims to advance knowledge for the development of new treatments and therapies for SUDs. Future directions in the SUDs field may include studies evaluating how different neural systems integrate, thorough analyses of intercellular communication and intracellular top-down mechanisms, and testing the causal mechanisms using translationally valuable designs.
